# Molecular characterization of *Cryptosporidium* spp., *Enterocytozoon bieneusi* and *Giardia duodenalis* in laboratory rodents in China[Fn FN1]

**DOI:** 10.1051/parasite/2022046

**Published:** 2022-10-11

**Authors:** Nanhao Wang, Ke Wang, Yufeng Liu, Xiaotian Zhang, Jinfeng Zhao, Sumei Zhang, Longxian Zhang

**Affiliations:** 1 College of Veterinary Medicine, Henan Agricultural University Zhengzhou 450046 Henan PR China; 2 International Joint Research Laboratory for Zoonotic Diseases of Henan Zhengzhou Henan PR China; 3 Key Laboratory of Quality and Safety Control of Poultry Products (Zhengzhou), Ministry of Agriculture and Rural Affairs Zhengzhou Henan PR China

**Keywords:** *Cryptosporidium* spp, *Enterocytozoon bieneusi*, *Giardia duodenalis*, Laboratory rodents, Molecular characterization

## Abstract

*Cryptosporidium* spp., *Enterocytozoon bieneusi* and *Giardia duodenalis* are significant zoonotic intestinal pathogens that can cause gastrointestinal symptoms such as diarrhea and induce a host immune response. A total of 1237 fecal samples were collected from laboratory rodents (rats, mice and guinea pigs) from four different locations in China to investigate the infection rates and molecular characterization of these pathogens on experimental animals. Genomic DNA was extracted from each sample, and PCR amplifications were done. Overall, the *Cryptosporidium* spp. infection rate was 3.8% (47/1237). Four known *Cryptosporidium* species were identified, namely *C. parvum*, *C. muris*, *C*. *tyzzeri* and *C. homai*, the three former being zoonotic species. The overall *E. bieneusi* infection rate was 3.0% (37/1237). Seven known *E. bieneusi* genotypes, namely S7, BEB6, J, Henan-IV, CHG10, D and WL6, were detected by sequence analysis. Among these, genotypes D, Henan-IV and CHG10 have a high zoonotic risk. *Giardia duodenalis* was not detected at any of the three loci (SSU rRNA, *bg* and *gdh*) after PCR amplification. This study provides basic data for these pathogens in laboratory rodents in China and lays the foundation for their prevention and control in laboratory animals.

## Abstract (fr)

*Cryptosporidium* spp., *Enterocytozoon bieneusi* et *Giardia duodenalis* sont des agents pathogènes intestinaux zoonotiques importants, qui peuvent provoquer des symptômes gastro-intestinaux tels que la diarrhée et induire une réponse immunitaire de l’hôte. Au total, 1237 échantillons fécaux ont été prélevés sur des rongeurs de laboratoire (rats, souris et cobayes) de quatre endroits différents en Chine pour étudier les taux d’infection et la caractérisation moléculaire de ces agents pathogènes sur des animaux de laboratoire. L’ADN génomique a été extrait de chaque échantillon et des amplifications par PCR ont été effectuées. Dans l’ensemble, le taux d’infection par *Cryptosporidium* spp. était de 3,8 % (47/1237). Quatre espèces connues de *Cryptosporidium* ont été identifiées, à savoir *C. parvum*, *C. muris*, *C. tyzzeri* et *C. homai*, les trois premières étant des espèces zoonotiques. Le taux global d’infection par *E. bieneusi* était de 3,0 % (37/1 237). Sept génotypes connus d’*E. bieneusi*, à savoir S7, BEB6, J, Henan-IV, CHG10, D et WL6, ont été détectés par analyse de séquence. Parmi ceux-ci, les génotypes D, Henan-IV et CHG10 ont un risque zoonotique élevé. *Giardia duodenalis* n’a été détecté à aucun des trois loci (SSU ARNr, *bg* et *gdh*) après amplification par PCR. Cette étude fournit des données de base sur ces agents pathogènes chez les rongeurs de laboratoire en Chine et pose les bases de leur prévention et de leur contrôle chez les animaux de laboratoire.

## Introduction

*Cryptosporidium* spp., *Enterocytozoon bieneusi*, and *Giardia duodenalis* are three common intestinal pathogens found both in humans and in a large number of animals throughout the world [27]. These three pathogens can cause clinical symptoms such as weight loss, malnutrition and diarrhea, can induce an immune response in the infected host [2, 34], and can even lead to the death of the host [6]. These pathogens mainly spread via the fecal–oral route and can also be transmitted through contaminated food or water [11].

To date, at least 45 valid *Cryptosporidium* species and approximately 120 genotypes have been described [10, 13, 15, 36]. In rodents, at least 25 *Cryptosporidium* species have been identified [33]. Among laboratory rodents, six *Cryptosporidium* species have been identified, including *C*. *tyzzeri*, *C. ubiquitum*, *C. muris*, *C. andersoni*, *C. wrairi* and *C. homai*. Of these, *C. tyzzeri*, *C. ubiquitum*, *C. muris* and *C. andersoni* are zoonotic [36]. However, there are few reports regarding *Cryptosporidium* spp. infection status in laboratory rodents.

*Enterocytozoon bieneusi* belongs to the phylum Microsporidia [21]. More than 500 *E. bieneusi* genotypes have been identified in humans and animals by polymorphism analysis based on the ribosomal internal transcribed spacer (ITS) [20], and they have been placed into 11 distinct groups (Groups 1–11) via phylogenetic analysis. More than 90% of the genotypes belong to Group 1 or 2 with Group 1 genotypes being highly zoonotic. To date, more than 80 genotypes have been identified in rodents (including companion, laboratory and wild rodents), among them BEB6, PigEbITS7, Henan-II, CHN4, Type IV, C, I, J, D, Peru11, Peru8, EbpA, EbpC, CZ3 and S6 can infect humans at the same time [9, 12, 28]. Scientists around the world have been studying the mechanism of infection and treatment of this pathogen [14]; however, the natural host, transmission route and the role of rodents in *E. bieneusi* transmission are still unclear.

Eight genetically distinct assemblages (A–H) of *G. duodenalis* have been defined, with zoonotic assemblages A and B found in both humans and animals. Host-adapted assemblages C and D are found primarily in dogs, E in ruminants, F in cats, G in rodents and H in seals [26, 31]. Among these, assemblages A, B and G are commonly detected in wild rodents [21].

Laboratory rodents are widely used in medical, biological, pharmacy, animal husbandry, veterinary medical and scientific research [17]. Whenever laboratory rodents are infected with *Cryptosporidium* spp., *E. bieneusi* or *G. duodenalis*, their physiological, biochemical and immunological indicators are affected, and the infection seriously interferes with test results and can potentially cause disease in both humans and animals [14]. Therefore, the purpose of this study was to determine the infection status and genotype distribution of each of these three intestinal pathogens in laboratory rodents in China and assess the public health potential of *Cryptosporidium* spp., *G. duodenalis* and *E. bieneusi* in laboratory rodents.

## Materials and methods

### Ethical standards

This study was conducted in accordance with the Chinese Experimental Animal Management Law of 1988. The research plan was approved by the Research Ethics Committee of Henan Agricultural University. Fecal samples of laboratory rodents were collected after the approval by the director of the Experimental Animal Center. No animals were injured during the fecal sample collection.

### Sample animal and specimen collection

**Figure 1:**
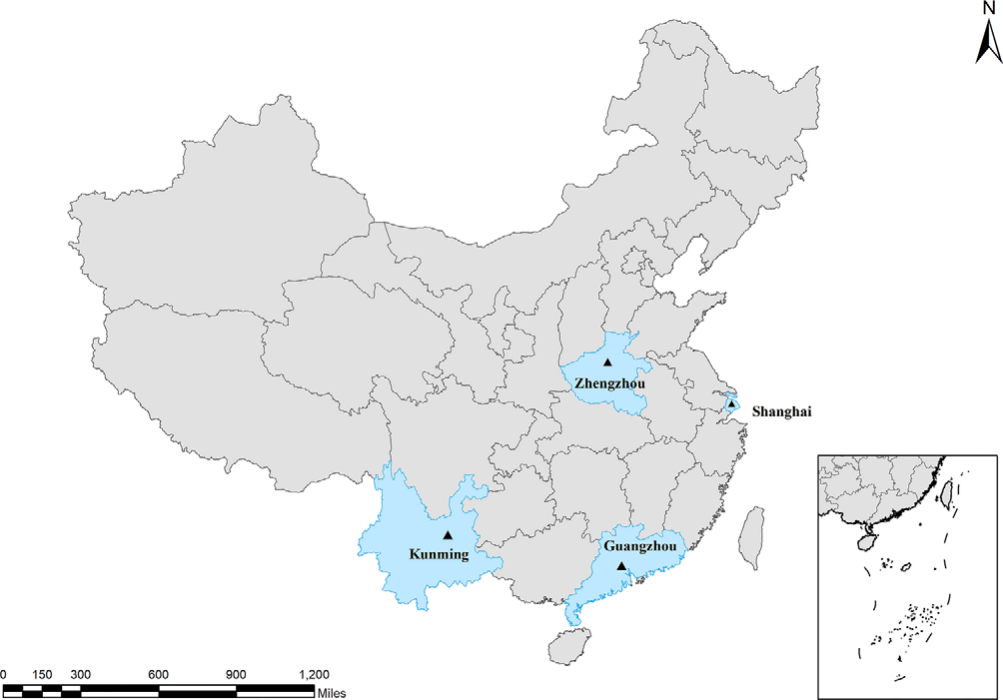
Sampling locations of the laboratory rodents in China. ▲: Sample collection site.

A total of 1237 fecal samples were collected between September 2019 and October 2020, from four medical experimental animal centers located in four different areas (300 from Zhengzhou, 150 from Kunming, 687 from Guangzhou and 100 from Shanghai) in China (Fig. 1). Sample distribution by animal host type was as follows: 118 samples from laboratory rats, 1027 samples from laboratory mice, and 92 samples from laboratory guinea pigs. These rodents belong to specific pathogen-free animals (SPF). The laboratory rodents were housed in individually ventilated cages (IVCs), each of which contained one to six animals. No obvious clinical symptoms were found when collecting feces. Approximately 2 g of fresh fecal samples were collected from each cage, placed in clean plastic zipper bags, marked with the pertinent information and shipped to the parasitology laboratory under cool conditions (4 °C) for further detection. Once in the laboratory, the specimens were stored in 2.5% potassium dichromate (4 °C) before undergoing molecular biology testing.

### DNA extraction

Total genomic DNA was extracted from each fecal sample (approximately 200 mg) using an E.Z.N.A.^®^ Stool DNA Kit (Omega Bio-Tek Inc., Norcross, GA, USA), according to the manufacturer’s recommended protocol. The extracted DNA was stored at −20 °C until PCR amplification.

### PCR amplification

*Cryptosporidium* spp*.* was examined by nested PCR amplification of an ~840 bp fragment of the small subunit (SSU) rRNA gene [32]. *Enterocytozoon bieneusi* was identified and genotyped based on the PCR amplification of an ~389 bp fragment of the ITS of nuclear ribosomal DNA [7]. The presence of *G. duodenalis* was identified and genotyped by the PCR amplification of an ~292 bp fragment of the SSU rRNA gene [3], an ~510 bp fragment of the β-giardin (*bg*) gene [16], and glutamate dehydrogenase (*gdh*) gene (~520 bp) [8]. Both the positive and negative controls were included in each PCR amplification, and the PCR amplification was performed at least three times per sample.

### Sequence and statistical analysis

The positive secondary PCR amplicons were sequenced bidirectionally by SinoGenoMax Biotechnology Co., Ltd. (Beijing, China). To determine the species and genotypes, the sequences obtained in this study were aligned with reference sequences downloaded from GenBank using Clustal X 2.1. All the nucleotide sequences have been submitted to GenBank at the National Center for Biotechnology Information (NCBI). Geographical locations and ages of rodents for each intestinal pathogen were analyzed using the chi-squared (χ^2^) test. *P*-values were considered to be statistically significant when <0.05. To infer the phylogenetic relationships of the detected samples, neighbor-joining (NJ) trees were constructed with the MEGA X program, based on the evolutionary distance calculated with the Kimura 2-parameter model. The reliability of these trees was assessed via bootstrap analysis of 1000 replicates.

### Nucleotide sequence accession numbers

The nucleotide sequences in this study have been submitted to the GenBank database (GenBank Accession No. OP102682–OP102686, OP103973–OP103978, OP104909).

## Results

Occurrence of *Cryptosporidium* spp., *E. bieneusi* and *G. duodenalis*

Among the 1237 fecal samples collected from laboratory rodents, 3.8% were *Cryptosporidium-*positive (47/1237). The infection rates for *Cryptosporidium* in the laboratory rodents in Zhengzhou, Kunming and Guangzhou were 4.3% (13/300), 12.0% (18/150) and 2.3% (16/687), respectively. No *Cryptosporidium* infection was detected in Shanghai. Statistical analysis showed that the *Cryptosporidium* infection rate was the highest in Kunming and was significantly different in different areas (χ^2^ = 35.85, *p* < 0.01). The infection rates were 3.3% (27/824) in laboratory rodents older than 3 months and 4.8% (20/413) in laboratory rodents younger than 3 months. There was no statistically significant difference in the *Cryptosporidium* infection rate between younger and older rodents (χ^2^ = 1.85, *p* > 0.05). The *Cryptosporidium* infection rates were 4.3% (44/1027) in laboratory mice, 0.8% (1/118) in laboratory rats, and 2.2% (2/92) in laboratory guinea pigs. There were no statistically significant differences in the infection rates of *Cryptosporidium* among different species of laboratory rodents (χ^2^ = 4.14, *p* > 0.05). The *E. bieneusi* infection rates were 6.6% (20/300), 1.2% (5/150) and 1.7% (12/687) in Zhengzhou, Kunming and Guangzhou, respectively. No *E. bieneusi* infections were detected in Shanghai. The *E. bieneusi* infection rate was highest in Zhengzhou, and *E. bieneusi* infection rates in the intestines of laboratory rodents were statistically significantly different in different areas (χ^2^ = 20.78, *p* < 0.01). The *E. bieneusi* infection rate was 3.9% (32/824) in laboratory rodents older than 3 months and was significantly higher than laboratory rodents younger than 3 months (1.2%; 5/413) (χ^2^ = 6.77, *p* < 0.01). The *E. bieneusi* infection rates were 1.8% (18/1027) in laboratory mice, 7.6% (9/118) in laboratory rats, and 10.9% (10/92) in laboratory guinea pigs. There was a statistically significant difference in the infection rate of *E. bieneusi* in different species of laboratory rodents (χ^2^ = 33.85, *p* < 0.01). *G. duodenalis* was not found in any of the samples (Table 1).

**Table 1:** Occurrence and genotypic distributions of *Cryptosporidium* spp. and *Enterocytozoon bieneusi* in certain laboratory rodents in China.

Items	No. samples examined	*Cryptosporidium* spp*.*		*Enterocytozoon bieneusi*		*Cryptosporidium* + *E. bieneusi*	
		Positive % (*n*)	Species (*n*)	Positive % (*n*)	Genotypes (*n*)	Positive % (*n*)	Species (*n*)
Locations							
Zhengzhou	300	4.3 (13)	*C. parvum* (3), *C. muris* (4), *C. tyzzeri* (4), *C. homai* (2)	6.6 (20)	D (1), BEB6 (2), Henan-IV (4), CHG10 (1), WL6 (2), S7 (10)	1.0 (3)	*C. tyzzeri* + Henan-IV (2), *C. tyzzeri* + BEB6 (1)
Shanghai	100	0	–	0	–	0	–
Guangzhou	687	2.3 (16)	*C. parvum* (6), *C. tyzzeri* (10)	1.7 (12)	BEB6 (6), J (6)	0.3 (2)	*C. tyzzeri* + BEB6 (2)
Kunming	150	12 (18)	*C. tyzzeri* (18)	1.2 (5)	BEB6 (5)	0	–
Species							
Rats	118	0.8 (1)	*C. tyzzeri* (1)	7.6 (9)	J (2), BEB6 (7)	0.8 (1)	*C. tyzzeri* + BEB6 (1)
Mice	1027	4.3(44)	*C. parvum* (9), *C. muris* (4), *C. tyzzeri* (31)	1.8 (18)	D (1), J (4), BEB6 (6), CHG10 (1), Henan-IV (4), WL6 (2)	0.4 (4)	*C. tyzzeri* + Henan-IV (2), *C. tyzzeri* + BEB6 (2)
Guinea pigs	92	2.2 (2)	*C. homai* (2)	10.9 (10)	S7 (10)	0	–
Age							
0–3 months	413	4.8 (20)	*C. parvum* (9), *C. muris* (4), *C. tyzzeri* (5), *C. homai* (2)	1.2 (5)	CHG10 (1), Henan-IV (3), BEB6 (1)	0	–
≥3 months	824	3.3 (27)	*C. tyzzeri* (27)	3.9 (32)	WL6 (2), D (1), S7 (10), J (6), BEB6 (12), Henan-IV (1)	0.6 (5)	*C. tyzzeri* + Henan-IV (2), *C. tyzzeri* + BEB6 (3)
Total	1237	3.8 (47)	*C. parvum* (9), *C. muris* (4), *C. tyzzeri* (32), *C. homai* (2)	3.0 (37)	D (1), BEB6 (13), Henan-IV (4), CHG10 (1), WL6 (2), S7 (10), J (6)	0.4 (5)	*C. tyzzeri* + Henan-IV (2), *C. tyzzeri* + BEB6 (3)

*Note*. –: No genotypes were found.

### Coinfection of enteric pathogens

Five samples were found to be positive for both *Cryptosporidium* and *E. bieneusi*, 0.4% (5/1237). Three *C. tyzzeri*-positive samples had coinfection with *E. bieneusi* genotype BEB6, and two samples had coinfection with *C. tyzzeri* and *E. bieneusi* genotype Henan-IV.

### Distribution of *Cryptosporidium* species

**Figure 2:**
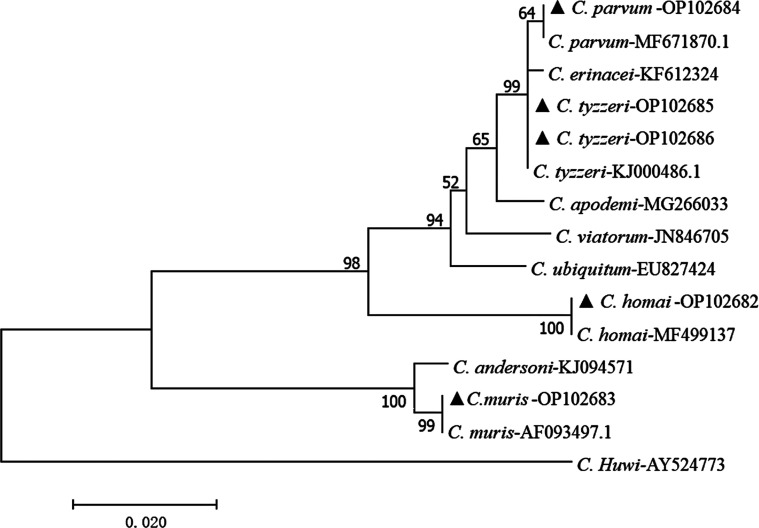
Neighbor-joining tree based on *Cryptosporidium* SSU rRNA Sequence. ▲: Species from this study.

Four *Cryptosporidium* species were detected: *C. parvum*, 0.7% (9/1237), *C. muris*, 0.3% (4/1237), *C. homai*, 0.2(2/1237), and *C. tyzzeri*, 2.6% (32/1237) (Table 1). *Cryptosporidium tyzzeri* is the dominant species. The distribution of *Cryptosporidium* in different species of rodents is different. Laboratory mice were infected with *C. parvum*, *C. muris*, and *C. tyzzeri*, only *C. tyzzeri* was found in laboratory rats, and *C. homai* was found in laboratory guinea pigs. *Cryptosporidium parvum*, *C. muris*, and *C. homai* were found in laboratory rodents younger than 3 months, *C. tyzzeri* were found in both younger than 3 months and older than 3 months animals. According to the sequence analysis, we found *C. parvum, C. muris* and *C. tyzzeri* with the risk of zoonosis (Fig. 2, Table 1).

### Distribution of *E. bieneusi* genotypes

**Figure 3:**
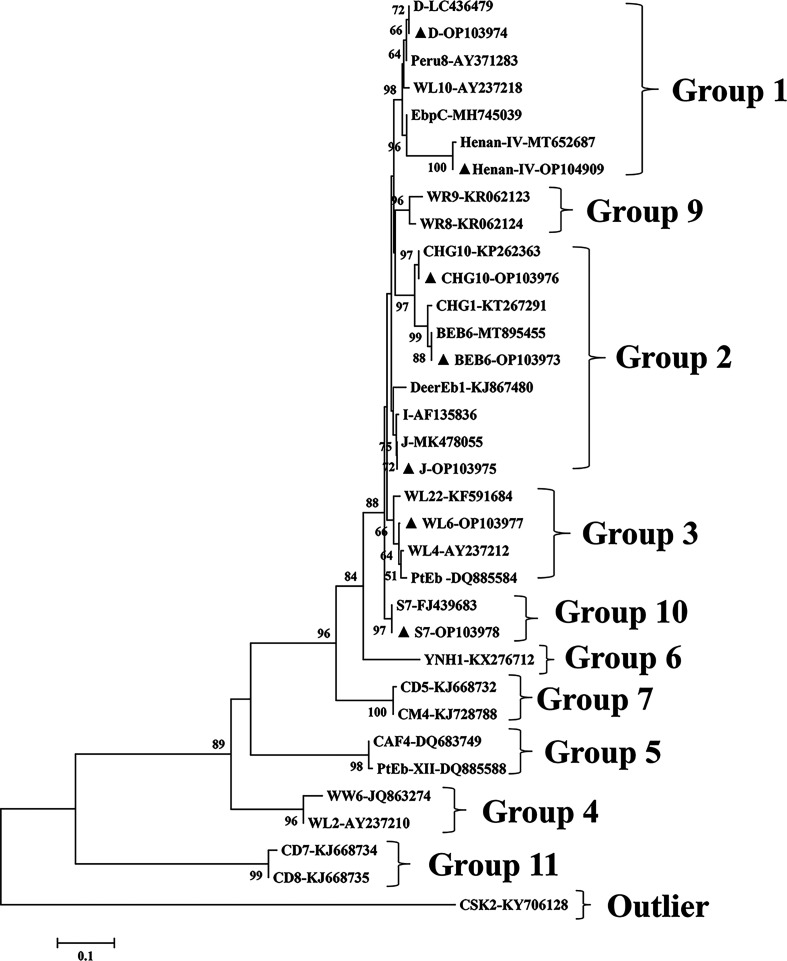
Neighbor-joining tree based on *E. bieneusi* ITS sequences. ▲: Genotypes from this study.

Thirty-seven specimens were positive for *E. bieneusi*, with a positive rate of 3.0%, and seven reported genotypes (namely, S7, BEB6, J, Henan-IV, CHG10, D and WL6) of *E. bieneusi* were identified, of which the infection rates of S7 and BEB6 were higher. Laboratory mice were infected with BEB6, Henan-IV, D, J, CHG10 and WL6, only S7 was found in laboratory guinea pigs, and J and BEB6 were found in laboratory rats. Genotypes D, Henan-IV and CHG10 were classified as Group 1 and had high zoonotic risk. Genotypes J and BEB6 were classified as Group 2 and had potential zoonotic risk. Genotypes WL6 were classified as Group 3, and Genotypes S7 were classified as Group 10 (Fig. 3, Table 1).

## Discussion

This study examined *Cryptosporidium*, *G. duodenalis* and *E. bieneusi* infection rates in certain species of laboratory rodents in China. *Cryptosporidium* is a common pathogen that infects rodents worldwide. The overall prevalence of *Cryptosporidium* in rodents was found to be 17% in one systematic summary [29]. In the current study, the overall infection rate of *Cryptosporidium* was 3.8% (47/1237), which is higher than the previously reported infection rates of 1.9% (5/264) and 0.6% (2/355) in laboratory rodents [21, 22]. According to a report, the infection rate of laboratory mice 1.7% (4/229) is lower than that of this study 4.3% (44/1027), and that of laboratory rats 4% (1/25) is higher than that of this study 0.8% (1/118) [22]. A report investigated the *Cryptosporidium* infection rate of laboratory rats in four regions in China, which was 0.6% (2/355), similar to the results of this study [21]. Many studies have reported *Cryptosporidium* infections in wild rodents all over the world. In previous studies, it is reported that the infection rate of wild rodents was 20.5% (3848/18,804) around the world, and it has been suggested that *Cryptosporidium* infection is more common in wild rodents than in laboratory rodents [36]. This may be because the organism is easily transmitted through environmental pollution under natural conditions.

In this study, four *Cryptosporidium* species were identified, among which *C. parvum*, *C. muris* and *C. tyzzeri* were zoonotic. These results are different from those reported in previous studies. In one study, only *C. tyzzeri* (formerly known as Mouse genotype I) was found in laboratory mice and rats [22]. In a study from Nigeria, *C. andersoni* and *Cryptosporidium* rat genotype II were identified in laboratory rats [4]. In another investigation, *C. ubiquitum* and an undetermined *Cryptosporidium* genotype were found in experimental rats in China. [19]. In Australia, *C. homai* was reported to be found in laboratory guinea pigs [35]. The results of this study show that *Cryptosporidium* infection in laboratory rodents presents a zoonotic risk and provide basic information for the prevention and control of *Cryptosporidium* in laboratory rodents. However, the positive samples for *C. parvum* failed to be sub-genotyped by the *gp60* marker to determine the genotypes within *C. parvum*.

There are few reports about *E. bieneusi* infection in laboratory rodents, showing significant differences in infection rates. In the current study, the *E. bieneusi* infection rate was found to be 3.0% (37/1237). The *E. bieneusi* infection rate was found to be 37.9% (11/29) in laboratory prairie dogs in the USA [24]. In a study carried out in China on 291 laboratory rat fecal samples, PCR amplification showed that 4.8% (14/291) were infected with *E. bieneusi*, which is lower than in this study [18]. The results of this study show that more research is needed on *E. bieneusi*.

In this study, seven known genotypes were identified in 37 samples. Genotypes J and D are common and have been reported in many countries and many animals. S7 has been reported in humans and bovines in the Netherlands and China [30]. BEB6 has been reported in chinchilla in China and in other animals in the USA, Brazil and Peru. Henan-IV and CHG10 have only been reported in China. WL6 has been reported in muskrats in the USA [28]. Previous studies have identified several known and previously unknown *E. bieneusi* genotypes. Four known *E. bieneusi* genotypes (namely, EbpA, EbpC, S7 and N) and a newly discovered genotype (SHR1) were found in laboratory rats in three cities in China [18]. Genotypes D (*n* = 1), Henan-IV (4) and CHG10 (*n* = 1) were classified as Group 1 and had high zoonotic risk. Twelve *E. bieneusi* genotypes were found in four species of wild mice in southwestern Poland: two known genotypes, D and new gorilla1, and genotypes WR1–WR10 [23]. Seven genotypes (namely, EpbA, C, D, H, PigEBITS5, CZ3 and Peru8) have been identified in wild house mice in the Czech–German border area, while only one (Row) has been reported in an American laboratory [24, 25]. Peru16 was found in guinea pig in Peru [9]. Two genotypes (D and Peru6) were found in wild rodents in Heilongjiang Province, and four (CHG14, BEB6, D and CHG2) were found in homologous rodents around dairy farms in Henan Province [37]. These studies identified different intestinal *E. bieneusi* genotypes in different animal hosts. In general, zoonotic genotypes, such as D, EbpA, EbpC, CHG9 and Pig EBITS7, were identified, indicating that intestinal *E. bieneusi* spreads between rodents and different animals.

*Giardia duodenalis* was not found in laboratory rodents in this investigation. However, a previous study reported a *G. duodenalis* infection rate of 9.3% (33/355) in laboratory rats [19]. The different detection rates in these surveys may be due to differences in animal species, age distribution, sample size, host health status, management level, and population density. Since data regarding *G. duodenalis* infection in rodents are limited, further epidemiological surveys involving various species of rodent hosts should be undertaken to better understand the genetic diversity, host specificity, and transmission modes of this parasite.

Laboratory animal centers usually have exceptional sanitary conditions and standards, with food and water sterilized prior to being given to animals [17]. The laboratory rodents in this study were fed purified water and pellet food that had been disinfected, and lived in an independent isolation environment; however, some laboratory rodents were still infected with *Cryptosporidium* and *E. bieneusi*. The laboratory rodents investigated in this test are SPF animals. According to the standard formulated by China (Gb/T 14922.2-2001), these three kinds of pathogens are not included in the test items; however, these pathogens are at risk of zoonotic diseases. There is no direct evidence of how these pathogens spread in laboratory rodents, and data on environmental samples from positive feeding facilities are lacking [1]. Therefore, environmental samples from feeding facilities should be further examined to understand possible transmission routes. Research also shows that laboratories should conduct parasite detection when using non-specific pathogen-free rodents, to improve the accuracy of experimental results and the safety of the experimenter. Laboratory rodents are usually asymptomatic after infection. However, the infection can cause bowel disorders even in rodents with no obvious symptoms [5], which can significantly impact experimental results. Frequent validation of purification for laboratory rodents and the environment is needed. In particular, attention should be paid to the disinfection and sterilization of feed, bedding and cages, as well as the sanitation of drinking water, so as to effectively control parasitic infection and improve the quality of experimental animals.

## Conclusion

This study indicates that the infection rates with *Cryptosporidium*, *E. bieneusi*, and *G. duodenalis* were low in laboratory rodents in China. These animals can be co-infected with *Cryptosporidium* spp. and *E. bieneusi*. Most of the species found in this study are zoonotic. Therefore, improving the management level, strengthening the monitoring and control of parasite infection, improving the feeding conditions and environmental settings, and strictly establishing the health management system for laboratory animals are necessary. A public education program on the infection potential of the three pathogens should be implemented, including breeders and laboratory personnel as part of the “one health” approach to the prevention and control of infection.

## Conflict of interest

The authors declare that there are no conflicts of interest.
